# Remarks on the Medical Topography of Callao and Lima

**Published:** 1850-12

**Authors:** G. R. B. Horner

**Affiliations:** Fleet Surgeon, Pacific Squadron


### *Remarks on the Medical Topography of Callao and Lima.* By G. R. B. Horner, M. D., Fleet Surgeon, Pacific Squadron.

On a fair day, the approach to these celebrated places is highly picturesque. The Andes are seen to the eastward and behind them, raising their snow capped summits thousands of feet above the mountains between the former and the ocean, and though formed of barren russet ash-like rocks, their sublimity, ruggedness, and variegation of outline render the prospect of them exceedingly striking. On approaching more nearly from the sea, we perceive to the south east, for miles, a high perpendicular and rocky shore, constantly washed and beaten down by the surf, and directly eastward the rugged precipitous island of del Fronton. Separated from this by a narrow strait is that of St. Lorenzo, which rises 1280 feet above the ocean, and forms the western side of the harbor of Callao. Between the two islands are perceptible the ships at anchor there, displaying their masts and banners above the low, sandy, pebbly peninsula, on which stand Callao and its immense castle. Between the western end of this peninsula and St. Lorenzo is the Bouqueron passage, but it is a dangerous one, from its rocks, shoals, and narrowness, and vessels generally run into the harbor through the northern entrance, between the upper end of St. Lorenzo and the main land. In doing this we have a magnificent view of the mountains, harbor, islands, Callao, Lima, and the country between it and the water, which though only in part cultivated, displays a luxuriance of vegetation, much in contrast with the barrenness of the mountains. As soon as a ship anchors she is surrounded by flocks of birds, feeding upon whatever they can gather upon or beneath the water. From morning to night, gulls, cormorants, ducks, and dark, bag-throated pelicans continue to swim, dive, and skim over the surface. Such flocks of them I have never seen in any other part of the world. But Callao presents little of interest; it contains only about 5000 souls, has irregular, mostly narrow, illy paved streets, and is built either of sunburnt bricks or adobes, plastered over, or of posts and reeds smeared over with mud. Recollection of the awful earthquake which caused the old town to sink beneath the ocean, and fear of another such catastrophe, prevent any attempt at style either in the size or structure of the houses. These are nearly all one story high, and flat roofed. The largest have inner courts, some of which are well painted, quite spacious and handsome. Water is furnished chiefly from two large bronze fountains, each made of basins gradually diminishing in size from the lowest to the highest. The market is in the large square, and is tolerably well supplied with fruit and vegetables, but fish, flesh, and fowl are not abundant, and the numerous black vultures perched on the houses and castle walls, look half famished and disconsolate from the scarcity of meat thrown away by the inhabitants. The castle occupies about ten acres of ground, is separated from the town by a moat forty feet wide, eight or ten deep, and encloses two large round terraces, a chapel, and barracks, now chiefly used as the custom house. Between the castle and the point looking towards the Bouqueron passage, lie the skeletons of the multitude who perished by the sword, famine, and pestilence during the late civil wars of Peru. So numerous were the dead, that their bodies were dragged out of the castle and thrown upon the beach to moulder, or serve for food to birds and beasts. For an incredible period the air of the town was tainted with the putrid exhalations. Subsequently and after the suppression of a mutiny in the garrison—as I was informed by an eye-witness—160 men were shot, hauled out in carts to a pit dug out of the town, were thrown within it and consumed with fire. But at this time the air of Callao is as pure as that of most similar towns, and though frequently damp from moisture, whole cargoes of Chilian wheat are kept constantly heaped in vast piles, upon the space between the town and the long quay erected by Gen. Miller. The wheat there lies white, clear, and plump for indefinite periods, though merely a few mats are thrown over it during the dampest weather. Callao is justly entitled to the appellation of salubrious, and furnishes little private practice to physicians, has no public hospitals, and only one private, that of Dr. Gallagher, of Lima. He pays it daily visits, and has Dr. Whittingham in constant attendance. The hospital is partly formed by the old custom house, stands on a declivity, a mile southeast of Callao, and adjoining the village of Bellavista. The location of the building is airy, convenient, and commanding. From it are seen the romantic country back of it, and likewise the town, harbour, ships, ocean, and the eastern face of Del Fronton and St. Lorenzo. The hospital has two courts, several rooms for offices, and three wards, large enough to accommodate fifty patients, and well ventilated by valves in the skylights over the middle of the wards. These are parallel to each other, open by doors into the chief court, are paved with bark, furnished with iron bedsteads and wool mattresses, and though not kept in the nicest order, are very comfortable for the poor weather-beaten sailors who resort to them for health. There were forty-three of them in the hospital when I visited it, and each one paid $1.50 per day for board and medical attendance. Officers are charged higher, and proportionately to their accommodations. The diseases in the hospital were those most common among seamen. An American had died in it very recently; and its walls had been shaken by two shocks of an earthquake the night before my visit, but they are thick, only a story high, and cannot be thrown down except by a very violent shock. We can then with good reason recommend the hospital to merchant seamen, and in emergencies to those of any vessels of war, crowded with sick, or having incurable and contagious cases, or such as would be benefitted by treatment on shore. Desertion is the strongest objection to sending men to this hospital, but that is trifling when we calculate the comfort afforded the sick, the better chances they would have of being cured, and the greater certainty of their not contaminating a whole crew by remaining on board ship.

From Callao to Lima is an inclined plain, six miles wide, rising gradually from one to the other, at the rate of eighty feet the mile. Lima then, is nearly five hundred feet above the ocean, and stands at the highest part, upon its eastern border, beneath a bare rugged ridge of granite. Through a gap of this flows the Rimac, a rapid mountain stream, rising from the Andes, and fed by their melted snows. The river runs through the northern end of the city, and when full is a hundred yards across, but almost all of its water is diverted from its channel to turn some mills, to supply the Limanians, and irrigate the country below the city. To effect this justly a commissioner is appointed to direct the water where wanted, and by aid of it and the dew the soil produces abundantly, as it is alluvial and naturally fertile. This is made manifest by its yielding two or three times a year crops of fruits and vegetables. Among its fruits are limes, lemons, oranges, pineapples, bananas, guavas, plums, pomegranates, the granadilla; the luscious fruit of a passion flower, the picaya, a very large legume with black seed and a sweet frost-like pulp, and the delicious cherimoyer. This is the fruit of a magnolia, is of a green color exteriorly, white within, has a scale-like rind, and contains many black seeds buried in a juicy, very sweet pulp, flavored slightly with strawberry. The cherimoyer is the most esteemed of Peruvian fruits, is wholesome and nourishing. It forms a chief article of food to the natives, and when it is of fullest size, will nearly yield enough nourishment for a breakfast. Of vegetables the most abundant are pumpkins, Indian corn, potatoes, cabbages, beans, and yuca, a species of mandiaca root, like that of Brazil, but used simply as a vegetable, and not made into farina nor tapioca. An ordinary method of cooking it, is to strip off its rusty, blackish skin and boil it with beef, with which it is eaten after soup has been served. The plant is cultivated in fields, attains a height of five or more feet, has a palmate leaf, and an irregularly fusiform root from one to two feet in length. The Limanians can boast of eating the best of potatoes, both sweet and common, or, more properly, indigenous as they were first found in Peru. The finest used are the large deep purple potatoes, and the small yellow, which are the most savory of the two kinds, and when well cooked have a delicacy, lightness, and gusto surpassing those of any other potatoes, not excepting the sweet of Lima, which are very good. These are generally of a deep yellow color inside, and differ from ours in shape by being knotted on the outside. Some of them are so much so that they resemble a common potato of great size, formed of five or six large tubers.

Of medicinal plants few are to be found wild or cultivated. The mountains are too rocky to produce them, the plains and valleys too much in demand for eatables, to allow them to grow abundantly. But on the banks of the Rimac above the city and near a mill race on the north side, the palma christi grows spontaneously, and perennially. Some of the nightshades are also produced, and among them the most remarkable is the Datura arborea, or Floripondio. It is strongly narcotic, thought dangerous in a bed chamber, attains a great height, and bears a perfectly white lily-like flower, composed of a single goblet-shaped petal eight inches long, with an everted margin, terminating in four points an inch or two in length.

To obtain a perfect view of the city and surrounding country, it is necessary to clamber to the top of St. Christopher, a mountain at its northern extremity. It is a part of the ridge mentioned, stands on the right side of the Rimac, rises seventeen hundred feet above the sea, and is formed of huge fissured granitic rocks, resembling the lava of Etna. Its summit has two points like the crater of Vesuvius, and on the highest a conical heap of stones and lime surmounted by a wooden cross. The ascent is steep, broken, and in parts slippery and dangerous, but the prospect beheld after the summit is attained fully compensates for fatigue suffered. Within the prospect are included the Andes, the valley of the Rimac, the bull circus, the new Alameda; a favorite public walk; the city, Callao, ocean, and its coast from above St. Lorenzo to that below Cherillos, the fashionable bathing place of the Limanians. From his airy position the spectator perceives that Lima, on the south side of the river, is hemmed in by a ditch and wall of adobes, except along its margin, and that it has rectangular streets of convenient width, that it abounds in churches and flat roofed dwellings, rarely more than a story high. But they occupy a great deal of ground, and have from one to two spacious courts included within their walls. These are formed of adobes, much larger than sun burnt bricks, but much smaller than the immense adobes composing the fences of the adjacent fields, for two of the latter kind, placed one above the other, are sufficiently high for an ordinary fence. The finest dwellings have very high apartments, opening into one another and the courts. On the back one of these are the kitchen and servants' chambers, and on the front those of the family, and the parlors. These are large, have wooden ceilings, plank or brick floors, and walls covered with paper, tacked on, as paste will not make it adhere to the adobes forming them. Chimneys are not used, and none are visible, though fire would be agreeable during the frequent drizzles which fall night and day, and are heavy enough to chill the air, make muddy streets and fill small hollows with water. The drizzles are called dews, but in the United States would be termed light rains. When they prevail, the Andes are entirely obscured in mist and clouds, and I have seen Fahrenheit's thermometer sink to 60° even in the harbor. But this is more properly to be ascribed to the ever blowing southerly trade winds, than to the dampness of the atmosphere, or the wide rapid streams from the Rimac, flowing in the middle of all the principal streets, and gushing from the basins of the bronze fountain in the great square or plaza. This has the viceroy's palace, now the president's, on one side, the bishop's and cathedral on another, and the two portals or arcades of shops on the remaining. The cathedral is a vast pile of adobes, lime, and wood, 245 feet in front, 375 in depth, and contains the remains of the first viceroy of Peru, Francisco Pizarro, but we will let them there rest in peace, and speak of the living Limanians. They are estimated at 85,000 by the last census, and consist of whites, blacks, mulattoes, Indians, and various admixtures. The officers of the government, those of the army, and the principal native families are of Spanish descent, the soldiers, hucksters, many of the servants, the market people of the city and country, are native Indians. Indeed in the four battalions composing the garrison, I did not see one who was not. They are copper colored, short and stout, rarely over five feet five inches high, often under that, and have low wide foreheads, black eyes and hair. Their physiognomy indicates mildness of temper, prudence, and a moderate amount of intelligence. They and all the other citizens are Roman Catholics, much addicted to festivals. One occurs besides the Sabbath every month for ten of the year, and two take place in each of the two remaining months. Idleness and dissipation are the natural consequences, and during these festivals the lower class of men consume a great quantity of spirit, chiefly pisco, so named from the town where it is manufactured. It resembles in color and taste corn whiskey, and is made from the fermented juice of the Peruvian grape. Lima has no good schools for extensive education, and only one medical. This has a full number of Professors, and eighty students, who have access to four hospitals in a limited manner. They are the female hospital of Santa Ana, the male one of St. Andros, the military, and incurable. The three first lie in the southern quarter of the city, the last is within the north eastern. They are all built similarly to the dwellings, and do not require minute description. It may be enough to state that they have a single story, are paved with brick, furnished with iron and plank bedsteads, and are indifferently well ordered.

The best hospital is St. Ana's. It has four large rooms forming a cross, with rooms between the limbs. It accommodates over 700 patients, has two courts and a female college adjoining, where 20 girls are educated gratis. St. Andres accommodates 400 patients, and like the military has a guard of soldiers to preserve order. The hospital of incurables is divided into a male and female department, with adjoining walls and courts, and contains altogether 80 patients. They are principally blacks and mulattoes, afflicted with chronic local disorders, mostly ulcers from leprosy and chigres, and presenting a most squalid appearance. In the other hospitals fevers and dysentery were two of the chief disorders. Among the former, intermittent is most common, as it is prevalent in the city, and has a fruitful source in the dampness of the houses, as well as in the heavy dews, if not in the exhalations of the neighboring country, borne over the city walls by the wind after it has swept across the bed of the Rimac and other low grounds between Lima and the Pacific. The two diseases above mentioned are probably the most common among the inhabitants, particularly the poorer classes who have unwholesome food, live mostly on fruits and vegetables, and occupy the dampest dwellings. The pitted faces of some, even of the fair sex, show that small pox is one of the diseases in the city, and the clumsy gait of some of the men makes known the existence of elephantiasis. Pulmonary affections mostly prevail when there is much humidity of the air. Catarrhs are very prevalent in winter when the city is both damp and dirty, and it is expressly prejudicial to asthmatics. Hence they become relieved by going into the country. Such, in fine, is the mortality of Lima from war and disease combined, that in spite of its many foreign and native physicians and its well filled drug stores, it is estimated that the whole number of deaths equals that of the population every seventeen pears. This estimate is extravagant, but the continuance of the city in precisely the same bounds it has had for a century, the erection of scarcely a single building in the year, the limited emigration of the people, the emptiness of the vast convent of St. Francisco, which forms three hollow squares, and the dwellings generally not being over crowded, prove the mortality to be great, and the vaults of the churches, the cells and graves of the Campo Santo to be abundantly supplied. Nevertheless, by preper care a person can live as long in this city as in any other, and while he lives can enjoy good society, a plentiful table, and pleasant promenades. He can resort to the mercantile reading room, the public library of 30,000 volumes, and the museum adjoining, where above the cabinets of animals, birds, beasts, coins, and Indian curiosities, are seen the full length portraits of Generals Gomorra, Lamar, and Bolivar, with those of the forty-five viceroys of Peru, from Pizarro in 1530, down to Laserna, the last one, who was expelled by the patriots in 1824. The museum is open gratis every day, except on the Sabbath and during festivals, and every person has the privilege of using the library, though no one is allowed to take the book away for private perusal.

